# Repeated successful vaginal delivery in a pregnant woman with unrepaired ectopia vesicae and split pelvis: a case study

**DOI:** 10.1186/s12884-020-02931-x

**Published:** 2020-05-12

**Authors:** Shaohua Liu, Xinhua Qu, Linlin Song, Ning Li, Aiqun Xu

**Affiliations:** 1 Department of Obstetrics, Yantai Affiliated Hospital of Binzhou Medical University, Yantai, 264000 China; 2 College of Clinical Medicine, Binzhou Medical University, Yantai, 264000 China

**Keywords:** Ectopia vesicae, Vaginal delivery, Split pelvis

## Abstract

**Abstract:**

Ectopia vesicae, or bladder exstrophy, is a rare malformation, more frequently found in males. Very few cases of pregnancy with unrepaired ectopia vesicae have been reported in literature. The majority of these pregnant women with ectopia vesicae have terminated their pregnancies by cesarean section due to malpresentation, preterm labor or other indications. Clemetson concluded that cesarean section was the preferable method of term delivery to avoid postpartum prolapse. We have a different opinion on this because we had an interesting case. A woman with unrepaired ectopia vesicae had two successful vaginal deliveries, in 2009 and 2019 respectively. She recovered well and did not have any symptoms or signs of pelvic organ prolapse (POP) so far.

**Case presentation:**

Let us present this woman with ectopia vesicae who had four pregnancies; two spontaneous abortions and two vaginal deliveries. In 2009, she had a successful vaginal delivery at Yantai Harbor Hospital where the first author worked at that time. She met the first author again surprisingly, during her third trimester in 2019. She had a spacious pelvis and pendulous abdomen. In this fourth pregnancy, the fetus changed its presentation frequently. Still, she had the second vaginal delivery successfully. She recovered fully after delivery and did not have any symptoms or signs of POP. As far as we know, this is the first case that a patient with ectopia vesicae who has been observed for such a long time after multiple vaginal deliveries.

**Conclusions:**

Doctors must evaluate the risk of vaginal delivery or cesarean section and consider maternal-neonatal health. Prior to this, women with repaired or unrepaired ectopia vesicae usually delivered their babies by cesarean section. Our practice shows that vaginal delivery is also a safe and feasible choice for some of these patients, especially for those with unrepaired, mild types of ectopia vesicae who experience no other dangerous or uncomfortable symptoms.

## Background

Ectopia vesicae, or bladder exstrophy, is a rare congenital malformation. Patients often have different degrees of split pelvis, deficiency of lower abdominal wall, abnormal development of umbilicus, epispadias, uterine malformation, vaginal septum, fistula, etc. [1–4] Split pelvis can also occur without other symptoms [5]. In the literature, it is reported more frequently in males than in females [6]. Reviewing the literature, there are several reports of pregnant patients. Clemetson [5] reviewed 64 pregnancies in 45 women between 1924 to 1958. Mandal [6] and Zimmer [7] each reported one case of ectopia vesicae both terminating in cesarean deliveries. We present a case with unrepaired ectopia vesicae who has experienced four pregnancies; two spontaneous abortions and two successful, full-term vaginal deliveries. Our case didn’t have any symptoms or signs of POP after her first vaginal delivery in 2009. To our knowledge, this is a unique case, that our patient has been observed for 11 years and after two successful vaginal deliveries. It has instructive significance in obstetric practice.

## Case presentation

A 19-year-old Chinese woman was admitted to Yantai Harbor Hospital on 14 September 2009 at 40 weeks of gestation, based on the last menstrual period [8]. Intermittent lower abdominal pains had started a few days prior to admission. She had an uneventful pregnancy course. This was her third pregnancy. She had two spontaneous abortions without apparent cause in 2008. Both lasted less than 60 days. Her menarche was at 14 and she was regular and bled for 3–4 days in a 28-day cycle. Other family members are all in good health.

On examination, her body temperature, pulse, respiratory rate, blood pressure were all within the normal ranges. She was a healthy-looking woman of medium stature and walked with a swing gait. Her height was 160 cm and she weighed 65 kg.

On abdominal examination, umbilicus was absent. There was a pigmentation area in the lower abdomen wall, about 8 cm in diameter. The pubic bones were separated widely. They were only connected by a tough fibrous band. There was no hair on mons pubis.

Upon an obstetric examination, the fundal height was consistent with term pregnancy. The fetal heart rate was 140 bpm. The vagina admitted two fingers, and the length was normal. There was transverse vaginal septum and only one cervix. Cervical length was about 3 cm. The external cervical os was closed, and the presenting part was the head. The abdominal wall was very thin and it was easy to touch the fetal body, just like an abdominal pregnancy.

A transabdominal ultrasound scan revealed that the fetus was consistent with 40 weeks of gestation with an estimated fetal weight of 3500 g. The amniotic fluid index was 15. Oxytocin and dinoprostone suppositories were administered in turn to induce uterine contractions. Four days later, she was finally in labor. The transverse vaginal septum was cut when the cervix dilated completely. A newborn baby girl weighing 3750 g with no obvious abnormalities was delivered, with Apgar scores of 10 at 1st and 5th minutes of life.

**Fig. 1:**
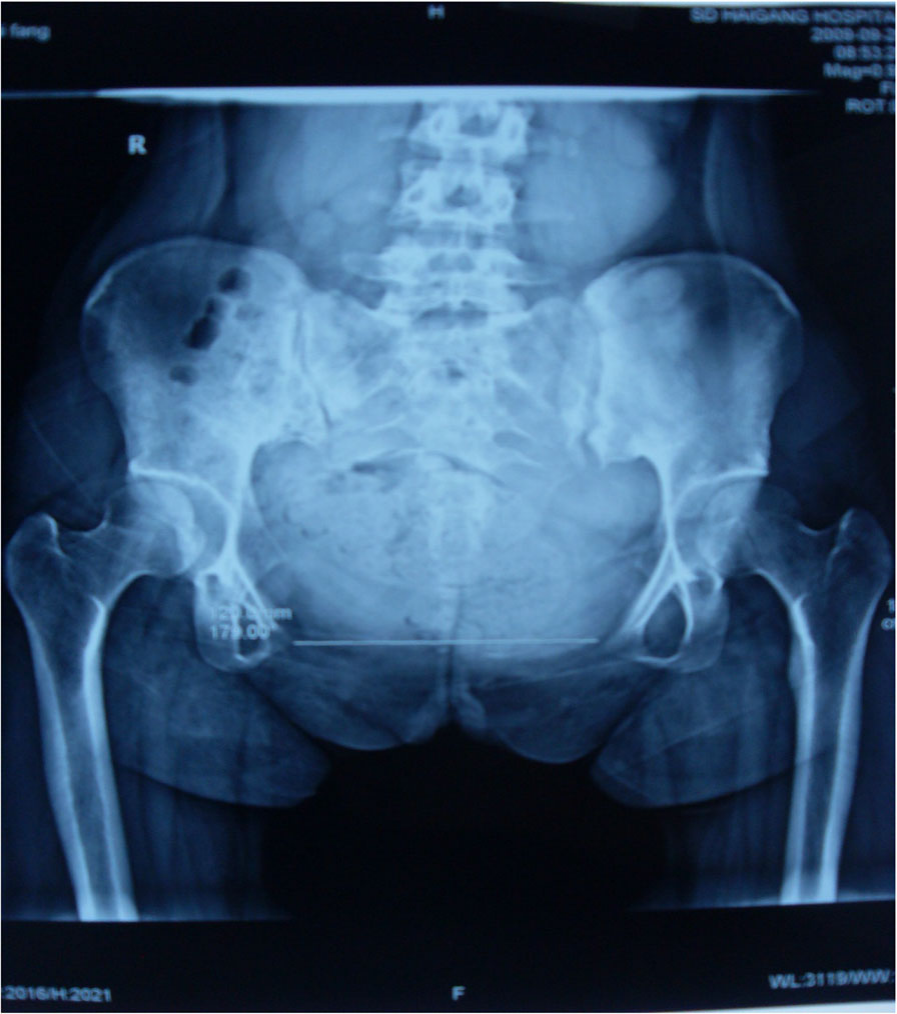
Front pelvic x-ray film-2009

A pelvis X-ray showed that the pubic symphysis was separated, the distance was 120 mm. (Fig. 1) After this pregnancy, she used an IUD for control birth, until April 2018.

She had the fourth pregnancy at the end of 2018. The pregnancy was uneventful until 28 weeks of gestation. It was a transverse lie. She tried to correct it by knee-chest position, because she wanted another vaginal delivery. It turned to breech presentation at 31 weeks. A transabdominal ultrasound scanning on bladder was carried out ahead of time, to prevent any trauma in case of cutting the thin abdominal skin. It showed that her bladder wall descended during micturition, and the lower abdomen wall was very thin, that you can even see the bladder wall descended under the skin. That meant there was no adhesion around the bladder. The fetus changed to vertex presentation at 38 weeks of gestation.

**Fig. 2:**
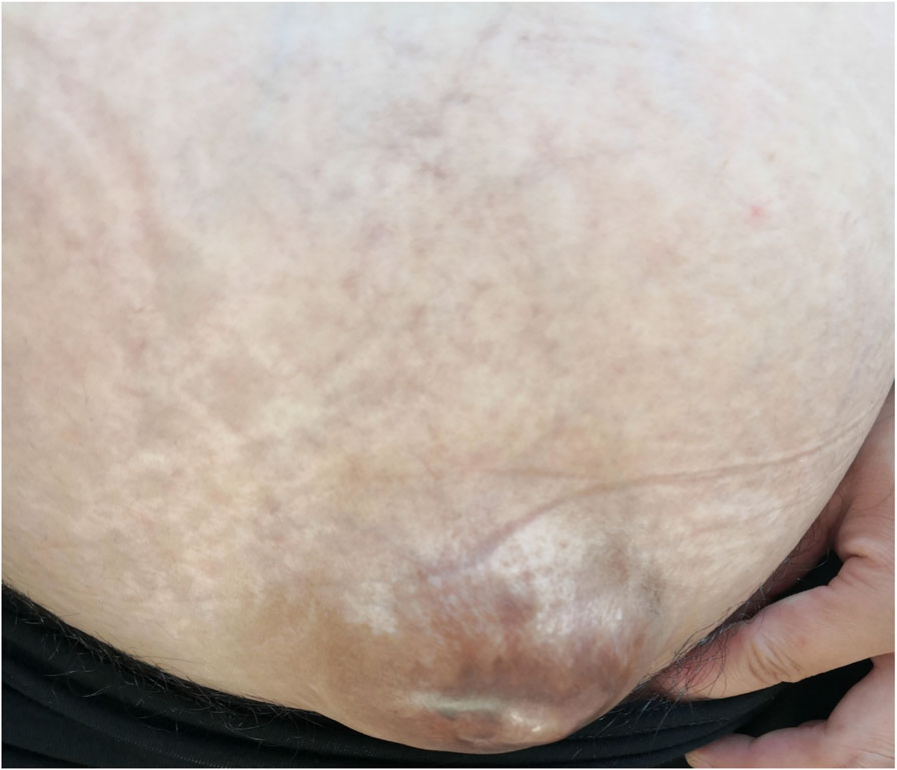
Stand before childbirth-2019

**Fig. 3:**
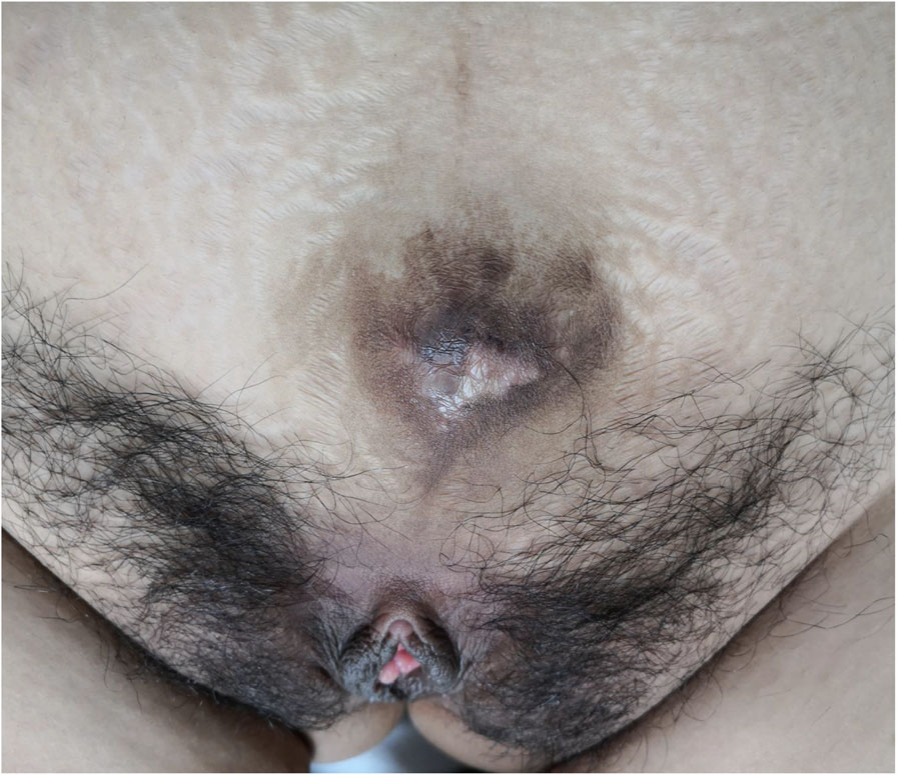
Lie on bed after childbirth-2019

She was admitted to our department at 39 weeks of gestation, based on the last menstrual period, due to premature rupture of membrane. Her bladder bulged forward through the thin lower abdominal wall much more obviously than that during the previous pregnancy. (Figs. 2 and 3) The fundus uteri were 10 cm below the xiphisternum and the uterus was broader than it was long, like shoulder presentation. A vaginal examination showed that the fetal head was not engaged, it lay in the left Iliac fossa. After pressing the uterus fundus, the fetal head entered the pelvic inlet obliquely, and the fetal ear could be touched. Then, by pressing the lower abdominal wall towards the pelvic inlet harder, it changed to normal engagement immediately. The fetal heart rate was within normal range. Spontaneous uterine contraction became stronger and stronger. Five hours later, the cervix was found fully dilated. A medio-lateral episiotomy was performed in order to protect the pelvic floor. A newborn baby girl weighing 3300 g was delivered, with Apgar scores of 10 at 1st and 5th minutes of life. Uroschesis occurred after birth and was cured after catheterization. The newborn was sent to the neonatal department due to hyperbilirubinemia. 5 days later, both mother and daughter were discharged in good general condition.

## Discussion and conclusions

Ectopia vesicae occurs in 1/20,000–50,000 deliveries [6, 9] with a male to female ratio of 2–6:1 [2, 10, 11]. The incidence of ectopia vesicae in females is about 1/125,000–250,000 deliveries [12]. But the difference may not be so accurate, because sometimes a girl was raised as a boy [5]. Prenatal diagnosis gives the parents an opportunity to determine whether to continue the pregnancy or not, because the long-term results in boys always ends up with sexual dysfunction [10]. Ultrasound and magnetic resonance imaging (MRI) have been found to be a good method to diagnose ectopia vesicae [13–16].

With the advent of antibiotics and surgical techniques, more females with ectopia vesicae live to reproductive age [5]. However, pregnant women with ectopia vesicae were rare, and few of these women terminated pregnancy vaginally. Our patient has the slightest degree of ectopia vesicae according to Champney’s classification. She did not accept any surgical repairing, because she didn’t have any uncomfortable symptoms. Now she has had two successful vaginal deliveries during the past 10 years.

Champney [5] describes four degrees of bladder extrophy:

I. The lowest or slightest degree of deformity tending to extroversion, where the pubic bones are separated but there is no fissure in the abdominal wall; the weak linea alba forms a hernial pouch containing the bladder.
II. The bladder is perfect but protrudes through a fissure in the abdominal wall.
III. A full deformity, where both the bladder and the abdominal walls are cleft.
IV. In the highest or greatest degree of deformity, the extroverted bladder is separated into two halves by an opening of the intestine.

Even with the slightest degree of deformity, our patient delivered two healthy girls by vaginal delivery. It was not so easy, especially for the last pregnancy. She experienced malpresentation, as most patients do [5]. In time, the fetus changed to vertex, mainly due to her having a spacious pelvis.

Why did we choose vaginal delivery mode? In fact, a literature review revealed a controversy (cesarean section vs vaginal delivery) in such women. In the reviewed literature [7, 17, 18], most women with repaired or unrepaired ectopia vesicae terminated pregnancies by cesarean section due to malpresentation or preterm labor. The main reason for cesarean section was to prevent utero-cervical or vaginal prolapse [5]. Krisiloff [19] has proven that a cesarean section does not always help to avoid the prolapse. He showed that vaginal delivery can be easier, due to pelvic anatomy conditions.

Our patient delivered vaginally twice. Compared with other women who terminated pregnancy by cesarean section, our patient recovered well, with the exception of urochesis during the second postpartum period. We optioned that vaginal delivery method was reasonable both times because she had concluded both pregnancy courses safely, and her deficient abdominal wall was not damaged by the pressure of the growing fetus and amniotic fluids. We supposed that her abdominal wall still had enough tension to bear the pressure of a second vaginal delivery. Furthermore, during the last labor the fetus changed its presentation frequently during the pregnancy and the presenting head descended well when pressing through the abdominal wall. We determined that the woman’s pelvis was spacious enough to bear her baby. We were prepared that even in a breech position we could still start with vaginal delivery, because it would be easy to push the head through the thin lower abdominal wall to resolve the entrapment of the fetal head. This case shows that some gravida with untreated congenital ectopia vesicae and split pelvis might also have an easier delivery vaginally and the labor stage might be very shorter.

Furthermore, the patient’s involution of uterus and pelvic floor was rather good. Our patient did not have urine incontinence or POP after either vaginal delivery, even after 11 years.

All previous references have reported pregnancy in women either untreated or with surgically reconstructed bladders, most of them terminated their pregnancies via cesarean section. We believe this case is unique. To our knowledge, this is the first case of repeat vaginal delivery after a long delay and it shows that vaginal delivery is a safe and feasible choice for these types of patients with unrepaired mild types of ectopia vesicae and split pelvis. Still, it is important to evaluate the risks of vaginal delivery or cesarean delivery together with a urologist [15] and ultrasound specialist when treating pregnant women with ectopia vesicae.

## Data Availability

Not applicable.

## Abbreviations

MRI: Magnetic resonance imaging
POP: Pelvic organ prolapse
